# Macrophages Are the Key Players in Promoting Hyper-Inflammatory Response in a Mouse Model of TB-IRIS

**DOI:** 10.3389/fimmu.2021.775177

**Published:** 2021-11-26

**Authors:** Lalit Pal, Raj Nandani, Pawan Kumar, Bharati Swami, Gargi Roy, Sangeeta Bhaskar

**Affiliations:** Product Development Cell-1, National Institute of Immunology, New Delhi, India

**Keywords:** TB-IRIS-tuberculosis-associated IRIS, macrophage, *Mycobacterium tuberculosis*, inflammation, HIV—human immunodeficiency virus

## Abstract

TB-IRIS is an abnormal inflammatory response in a subset of HIV-TB co-infected patients shortly after initiation of anti-retroviral therapy (ART). Therapy in these patients could have greatly improved the life expectancy as ART reconstitutes the function and number of CD4+ T cells and many patients see improvement in symptoms but paradoxically up to 54% of co-infected patients develop TB-IRIS. Different studies have indicated that both innate and adaptive immunity are involved in the pathology of IRIS but the role of macrophages in abnormal activation of CD4+ T cells is poorly understood. Since macrophages are one of the major antigen-presenting cells and are infected by M.tb at a high frequency, they are very much likely to be involved in the development of TB-IRIS. In this study, we have developed a mouse model of experimental IRIS, in which M.tb-infected T-cell knockout mice undergo a fatal inflammatory disease after CD4+ T cell reconstitution. Lung macrophages and blood monocytes from M.tb-infected TCRβ^−/−^ mice showed upregulated expression of cell surface activation markers and also showed higher mRNA expression of inflammation-associated chemokines and matrix metalloproteases responsible for tissue damage. Furthermore, cytokine and TLR signaling feedback mechanism to control excessive inflammation was also found to be dysregulated in these macrophages under lymphopenic conditions. Previous studies have shown that hyperactive CD4+ T cells are responsible for disease induction and our study shows that somehow macrophages are in a higher activated state when infected with M.tb in an immune-deficient condition, which results in excessive activation of the adoptively transferred CD4+ T cells. Understanding of the mechanisms underlying the pathophysiology of TB-IRIS would facilitate identification of prospective biomarkers for disease development in HIV-TB co-infected patients before starting antiretroviral therapy.

## Introduction

HIV-M.tb co-infection has been the most challenging concern in the efforts to scale up antiretroviral therapy (ART) because a subset of patients develop a condition called TB-IRIS, which arises as M.tb-specific CD4+ T cells re-emerge after ART initiation (1). TB-IRIS is characterized by a hyperactive anti-mycobacterial immune response and occurs in diverse manifestations including fever, lymph node enlargement, abdominal lymphadenopathy, pulmonary or pericardial effusions, and brain tuberculoma (2, 3). According to a meta-analysis done by Muller’s group, it affects about 16% of HIV-TB co-infected patients after ART initiation (4). However, some studies have reported a higher percentage of TB-IRIS (54.2%) in India (5). There are two forms of TB-IRIS, “paradoxical” and “unmasking”; in paradoxical TB-IRIS, before ART initiation, HIV patients have confirmed diagnosis of TB with a positive response to anti-mycobacterial therapy. After initiation of ART, patients experience worsening of TB symptoms like lymphadenopathy and radiographic damage (6, 7). In unmasking TB-IRIS, there is no diagnosis and no anti-tuberculosis therapy before start of ART, and the appearance of pathology associated with TB is recognized only following ART because of the development of IRIS (8). Immunopathological basis of IRIS is still not fully understood and there are no biomarkers to predict which patient will develop this syndrome. Macrophages are the major host cells exploited by M.tb for their growth and duplication (9). M.tb survive inside the macrophages in maturation-arrested phagosomes followed by recruitment of M.tb-specific T lymphocytes and other immune cells, which result in granuloma formation (10, 11). Th1-polarized CD4+ T lymphocytes are potent IFN-γ producers and crucial for the elimination of intracellular M.tb from these cells as IFN-γ is the central factor in the activation of anti-mycobacterial activities of macrophages (12, 13). A couple of studies have suggested that dysregulated macrophage function in the CD4+ T lymphocyte-deficient hosts could be responsible for the development of TB-IRIS in patients (14, 15, 16). Monocytes produced increased level of pro-inflammatory cytokines in TB-IRIS patients compared with pre-IRIS controls (14). Notwithstanding these research studies, mostly done with peripheral blood compartment of humans, the underlying mechanisms mediating TB-IRIS remain to be fully elucidated.

Our study has provided evidence of the important role of macrophages in the development of hyperactive CD4+ T cell response and pathogenesis of TB-IRIS. Effect of T-cell deficiency on macrophage function and the underlying mechanisms in the context of M.tb infection have been analyzed. We have also described an animal model of TB-IRIS, i.e., M.tb-infected TCRβ^−/−^ mice that lack T cells and represent a similar condition like AIDS patients (in which, however, CD8+ T cells are present). Intravenous adoptive transfer of CD4+ T cells into M.tb-infected TCRβ^−/−^ mice resulted in rapid weight loss, low body temperature, and mortality. Both *ex vivo* and *in vivo* studies provide evidence that infected macrophages from TCRβ^−/−^ mice are hyperactivated, secrete higher amount of proinflammatory cytokines, and express higher level of cell surface activation markers and MHC-II as compared to M.tb-infected WT mice. Lung macrophages from M.tb-infected TCRβ^−/−^ mice after transfer of CD4+ T cells showed higher mRNA expression of inflammation-associated chemokines, matrix metalloproteases, and Toll-like receptors. Furthermore, cytokine and TLR signaling feedback mechanisms were also found to be dysregulated in macrophages in T-cell-deficient conditions, which resulted in excessive inflammation. These observations provide evidence that macrophages from TCR β^−/−^ mice are differently activated and communication between these macrophages and CD4+ T cells could be a key factor for the excessive inflammation and host tissue damage during mycobacterial IRIS.

## Materials and Methods

### Ethical Approval

The study protocol was approved by the Animal Ethics Committee of the National Institute of Immunology (IAEC#443/17). Experimental procedures followed the guidelines of the Animal Ethics and Biosafety Committee of the National Institute of Immunology (New Delhi, India).

### Animals

Inbred T-cell-deficient TCRβ^−/−^, wild-type C57Bl/6, and congenic CD45.1 mice (6–8 weeks) were maintained in the germ-free condition in the animal experimental facility at the National Institute of Immunology according to the guidelines for laboratory animals.

### Mycobacterial Culture and Infection in Mice

*Mycobacterium tuberculosis* (strain H37Rv and H37Rv-GFP) was grown in shaking conditions (120 RPM) at 37°C. 7H9 culture media (Difco™ Middlebrook) was prepared in sterile water following the manufacturer’s instruction. Culture media was supplemented with 0.1% tween-80, 10% Albumin Dextrose Catalase (ADC), and 0.2% glycerol. At OD 0.6, M.tb culture was harvested by centrifugation at 3,000 RPM for 15 min. M.tb infection (200 CFU/mice) was given by aerosol route using Madison inhalation exposure system (Madison Industries, USA) in the BSL-3 facility at Tuberculosis Aerosol Challenge Facility of International Centre for Genetic Engineering and Biotechnology (ICGEB).

### Isolation of Peritoneal Macrophages, M.tb Infection, and Co-Culture With CD4+ T Cells

Thioglycolate (4%) injection (1.5 to 2 ml) was given intraperitoneally, and after 72 h, the mice were sacrificed and injected with chilled PBS in the peritoneum. The peritoneal exudate was collected and seeded onto 6-, 12-, 24-, and 96-well tissue culture plates (for different experiments) in RPMI media with 10% fetal bovine serum (FBS) for 2–3 h to allow macrophages to adhere to the plates. Subsequently, non-adherent cells were removed by washing twice with RPMI. Macrophages were infected with M.tb at 10× multiplicity of infection (MOI) for 4 h. After infection, the cells were extensively washed twice with PBS to remove non-phagocytosed bacteria and purified CD4+ T cells were added to the culture at 1:2 ratio. Cells were incubated for 24/48 h before further analysis.

### Bone Marrow-Derived Macrophages

Bone marrow from femurs of C57Bl/6 WT and T-cell-deficient TCRβ^−/−^ mice (6–8 weeks) mice were dispersed and cultured in RPMI medium [containing 100 U/ml of penicillin and supplemented with 10% FBS and 20 ng/ml of mouse macrophage colony stimulating factor (Miltenyi Biotec)] at 37°C and 5% of CO_2_. Cells were cultured till 90% of cells were positive for CD11b (a monocyte/macrophage-specific marker) and were harvested after 7 days.

### CD4+ T Cell Purification and Adoptive Transfer

CD4+ T cells were isolated from the spleen of CD45.1 (C57Bl/6) WT mice and enriched by magnetic cell sorting using MACS magnetic beads (Miltenyi Biotec) as per the protocol given. The purity of CD4+ T cells as determined by FACS was found to be 90%–95% (**Figure S1**). The enriched population of CD4+ T cells was stained with 50 µM carboxy fluorescein succinimidyl ester (CFSE) before adoptive transfer. 2 × 10^6^ enriched CD4+ T cells were then injected intravenously in M.tb-infected C57Bl/6 WT and T-cell-deficient TCRβ^−/−^ mice.

### FACS Analysis of Cell Surface Markers

Flow cytometry assay was done to check the expression of specific cell surface markers (MHCII, CD80, CD86, and CD40) on macrophages. For surface staining, 1 × 10^6^ cells from each group were taken and stained with specific antibodies for cell surface markers (MHCII-APC, CD80-FITC, CD86-PE, F4/80-PECy7, and CD4-FITC) and incubated for 30 min at 4°C. Cells were washed twice in FACS wash buffer after incubation, then resuspended in FACS buffer, and flow cytometric analysis was done (FACS Verse BD Biosciences, USA).

### Preparation of Single-Cell Suspension from Lungs and Isolation of Macrophages

Mice were euthanized by using a Ketamine–Xylazine cocktail (2 mg ketamine + 0.2 mg xylazine/20 g of mice). Lung tissue was collected and chopped with a fine blade in RPMI. The chopped tissues were transferred to digestion enzyme cocktail of hyaluronidase (200 μg/ml), collagenase-A (700 μg/ml), and DNase (30 μg/ml) and incubated at 37°C for 45 min in shaking condition. To get a single-cell suspension, the extracellular matrix was removed by passing the digested lung tissue through a cell strainer (pore size 40 μm). RBCs were lysed using BD Pharm lyse solution. After RBC lysis, cells were allowed to adhere in T75 culture flask for 5 h; non-adherent cells were removed by washing twice with PBS. Culture flasks were maintained on ice throughout washing and tapped firmly in between. After washing, strongly adherent cells (mainly macrophages) were removed by using Zymefree (non-enzymatic cell dissociation solution from Himedia). Purity of these cells were determined by flow cytometry. About 80% of cells were CD11b^+^, CD14^+^, and F4/80^+^ (**Figure S6**).

### Estimation of Secretory Cytokines

The level of TNF-α, IL-12, IL-6, IFN-γ, IL-2, IL-10, IL-1β, IL-4, and IL-17 cytokines in the serum and peritoneal macrophages culture supernatants was quantified by cytokine beads array (BD Biosciences, San Jose, CA, USA) as per the manufacturer’s protocol.

### Nitric Oxide Measurement

The level of nitric oxide in serum and peritoneal macrophage culture supernatant was determined indirectly by measuring nitrite concentration using Griess reagent [1% sulfanilamide in 5% H_3_PO_4_ and 0.1% naphthyl ethylene diamine in distilled water] according to the manufacturer’s protocol. One hundred microliters of culture supernatants/serum was transferred into 96-well plates and then 100 μl of Griess reagent was added, and absorbance was measured at 540 nm.

### mRNA Expression Analysis

For mRNA isolation, lung macrophages were isolated according to the protocol mentioned in *Material and Methods section*. mRNA expression of relevant chemokines in lung macrophages from different groups of mice was determined by qRT-PCR. RNA was isolated from lung macrophages using One Step RNA Reagent (RNAiso Plus, Takara) as per the manufacturer’s protocol. The quantification of mRNA was done using a spectrophotometer and agarose gel electrophoresis was used to determine the quality of mRNA. One microgram of mRNA was used to prepare cDNA using cDNA Synthesis Kit (Revert Aid First Strand, Thermo Scientific, USA) according to the protocol. Real-time PCR was set up using the cDNA and primers specific to each gene, using an RT-PCR master-mix (TB Green Premix Ex Taq Takara). The 2^−ΔΔCt^ method was used to determine the relative quantities of these genes and were normalized with respective genes from uninfected control group.

### Evaluation of Phagosome–Lysosome Fusion

Peritoneal macrophages were seeded on UV sterilized coverslips in a 12-well plate, and M.tb-GFP infection was given for 4 h. Then, extracellular bacteria were removed by washing (twice with PBS), and culture was incubated in a CO_2_ incubator for 24 h at 37°C. After incubation, cells were then fixed overnight at 4°C in 1% paraformaldehyde followed by washing with PBS twice. Lysosomal compartment of the cells was stained by using 100 nM LysoTracker Red for 30 min at 37°C followed by DAPI staining. M.tb-GFP inside the lysosome was observed under a confocal microscope. In each category, a total of 50 fields were analyzed, M.tb-GFP outside lysosomes (green spots) and M.tb-GFP inside the lysosome (yellow spots) were counted.

### CFU Enumeration

Whole lungs were isolated from M.tb-infected mice at different time points, suspended in 1 ml of PBS and homogenized using PolytronR PT 1600 homogenizer (Kinematica, Lucerne, Switzerland). One hundred microliters of lung homogenates were diluted serially, plated in triplicates on 7H11 agar plates, and incubated for 3 to 4 weeks at 37°C. After incubation, bacterial colonies were counted. Similarly, bacterial load in the spleen and liver were determined by counting the number of colonies at different time points.

### Histology

Collection of lung lobes was done aseptically after systemic perfusion with PBS, fixed overnight with 4% w/v paraformaldehyde, embedded in paraffin, and sectioned to 2-μm tissue sections. The tissue sections were stained with hematoxylin–eosin.

### Surface and Core Body Temperature

Core body temperature was measured using a rectum probe, and for surface body temperature, an infrared camera thermometer (seek thermal camera) was used.

### Statistical Analysis

Prism7 (GraphPad, San Diego, CA, USA) was used for statistical analysis. Data were compiled as mean ± SEM/standard deviation (SD). Comparison between the groups was carried out using two-way analysis of variance (ANOVA). Significance was determined using the Holm-Sidak method. Tukey’s correction was used for multiple comparisons. Any *p*-value less than 0.05 was considered to be statistically significant.

## Results

### CD4+ T Cell Transfer into M.tb-Infected TCRβ^−/−^ Mice Causes Rapid Weight Loss, Fall in Body Temperature, and Mortality

**Figure 1:**
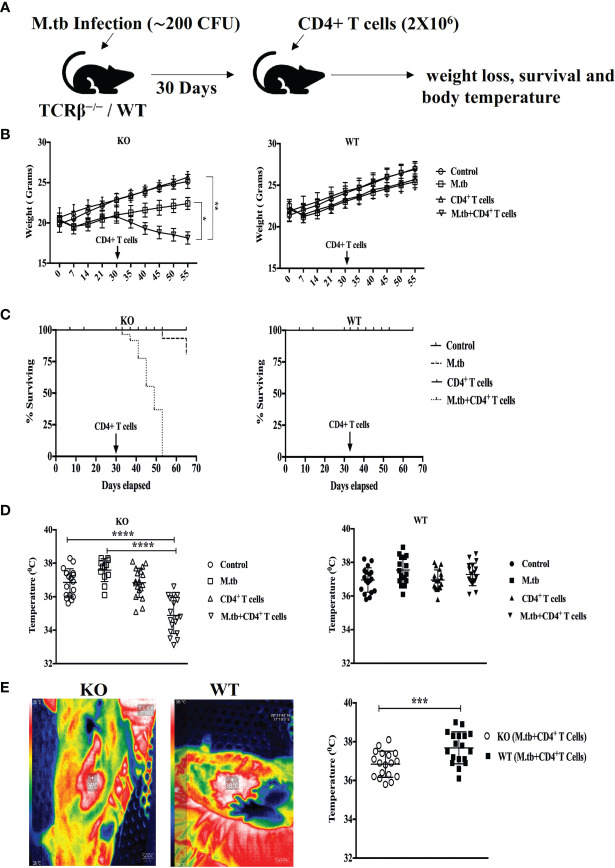
Adoptive transfer of CD4+ T cells in M.tb-infected TCRβ^−/−^ mice results in rapid weight loss, mortality, and low body temperature. **(A)** TCRβ^−/−^ and WT mice were challenged with M.tb (H37Rv) by aerosol route. After 30 days of infection, 2 × 10^6^ CD4+ T cells isolated from M.tb-infected WT CD45.1 (C57BL/6) mice were adoptively transferred to KO/WT mice. **(B, C)** Mice were followed for weight loss and survival (*n* = 8 mice/group). **(D, E)** Core body and surface body temperature were measured using rectum probe and thermal camera, respectively, 15 days post CD4+ T-cell transfer. Data are representative of six independent experiments. Statistical significance was determined by two-way ANOVA (Holm-Sidak method). *p < 0.02, **p < 0.01, ***p < 0.0010, ****p < 0.00001 KO, Knockout; WT, Wild type.

To investigate the effect of adoptive CD4+ T cell transfer from M.tb-infected CD45.1 (C57Bl/6) mice into TCRβ^−/−^ mice during chronic mycobacterial infection, TCRβ^−/−^ and C57Bl/6 WT mice were challenged with M.tb (about 200 CFU/mice) by aerosol route (**Figure 1A**). After 30 days of infection, enriched CFSE-labeled CD4+ T cells (2 × 10^6^) from M.tb-infected CD45.1 mice were injected intravenously into TCRβ^−/−^ mice and C57Bl/6 WT control mice. Recipient TCRβ^−/−^ mice rapidly lost 20%–30% of their initial body weight within 15 days of CD4+ T-cell transfer (**Figure 1B**). All the TCRβ^−/−^ mice in which CD4+ T cells were adoptively transferred succumbed by day 25 (**Figure 1C**). In contrast, infected C57Bl/6 WT recipient mice showed no significant weight loss and mortality. Core body and surface body temperature were also checked at different time intervals post-infection and after CD4+ T cell transfer. After M.tb infection, both groups showed increased body temperature, but after 15 days of CD4+ T cell transfer, TCRβ^−/−^ mice showed a significant drop in their core and surface body temperature (**Figures 1D, E**). This drop in core and surface body temperature could be because of heightened inflammation as part of the body’s natural defense (17). These results suggest that adoptive transfer of M.tb primed CD4+ T cells into M.tb-infected mice lacking T cells is sufficient to drive TB-IRIS, resulting in rapid wasting, excessive inflammation, and death.

It is reported that when CD4+ T cells are introduced in a T cell-deficient environment, they proliferate spontaneously (18). This observation was confirmed in our mouse model of TB-IRIS. Purified CD4+ T cells [cell purity was assessed by flow cytometry and 90%–95% of cells were found CD4 positive (**Figure S1**)] were labeled with CFSE. *In vivo* proliferation of CFSE-labeled CD4+ T cells from CD45.1 mice transferred to M.tb-infected KO as well as WT mice was studied. After 72 h of transfer, the CFSE dilution of CD4+ T cells in M.tb-infected KO and WT mice blood was compared. Higher dilution of CFSE was observed in KO mice providing evidence of higher proliferation of transferred T cells in M.tb-infected KO mice as compared to WT mice (**Figure S2**)]. These observations confirm that in this TB-IRIS model, proliferation of donor T cells in T-cell-depleted conditions is significantly high as compared to WT mice.

### Pulmonary/Systemic M.tb Burden and Lung Tissue Pathology of TCRβ^−/−^ Mice After Adoptive Transfer of CD4+ T Cells

**Figure 2:**
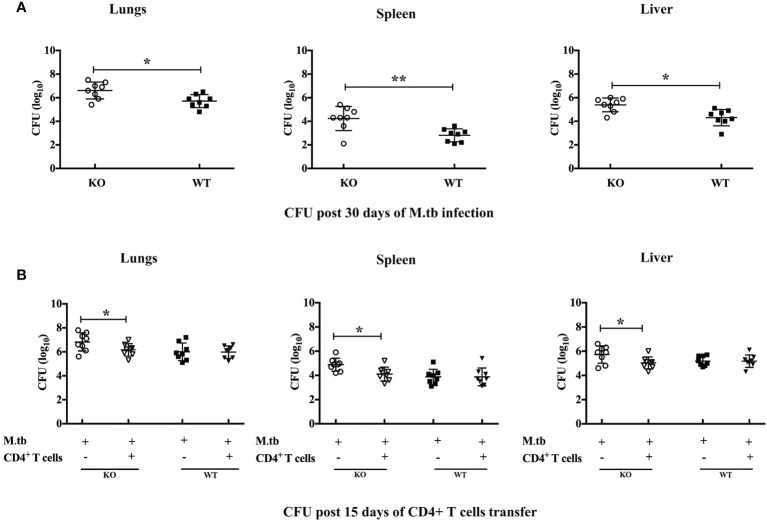
Pulmonary and systemic M.tb burden of TCRβ^−/−^ and WT mice after adoptive transfer of CD4+ T cells. Bacterial burden was measured in lung, spleen, and liver **(A)** 30 days post-infection and **(B)** 15 days post CD4+ T-cell transfer. Data are mean ± SEM of observations of three sets of experiments. *p < 0.01, **p < 0.001.

To investigate how efficiently these TCRβ^−/−^ mice control the M.tb infection, we compared the bacterial burden in lung, spleen, and liver in TCRβ^−/−^ and WT mice. In contrast to wild-type mice, TCRβ^−/−^ mice showed at least one log higher bacillary load in the lung, spleen, and liver at day 30 (**Figure 2A**). Interestingly, the transfer of CD4+ T cells into TCRβ^−/−^ mice significantly reduced the pulmonary and extrapulmonary bacterial burden while no such reduction was observed in WT mice. After 15 days of CD4+ T cell transfer, the bacterial burden in TCRβ^−/−^ animals was reduced by one log and it became similar in KO and WT mice (**Figure 2B**). This could be due to the hyper immune response in TCRβ^−/−^ mice, which killed the bacteria.

**Figure 3:**
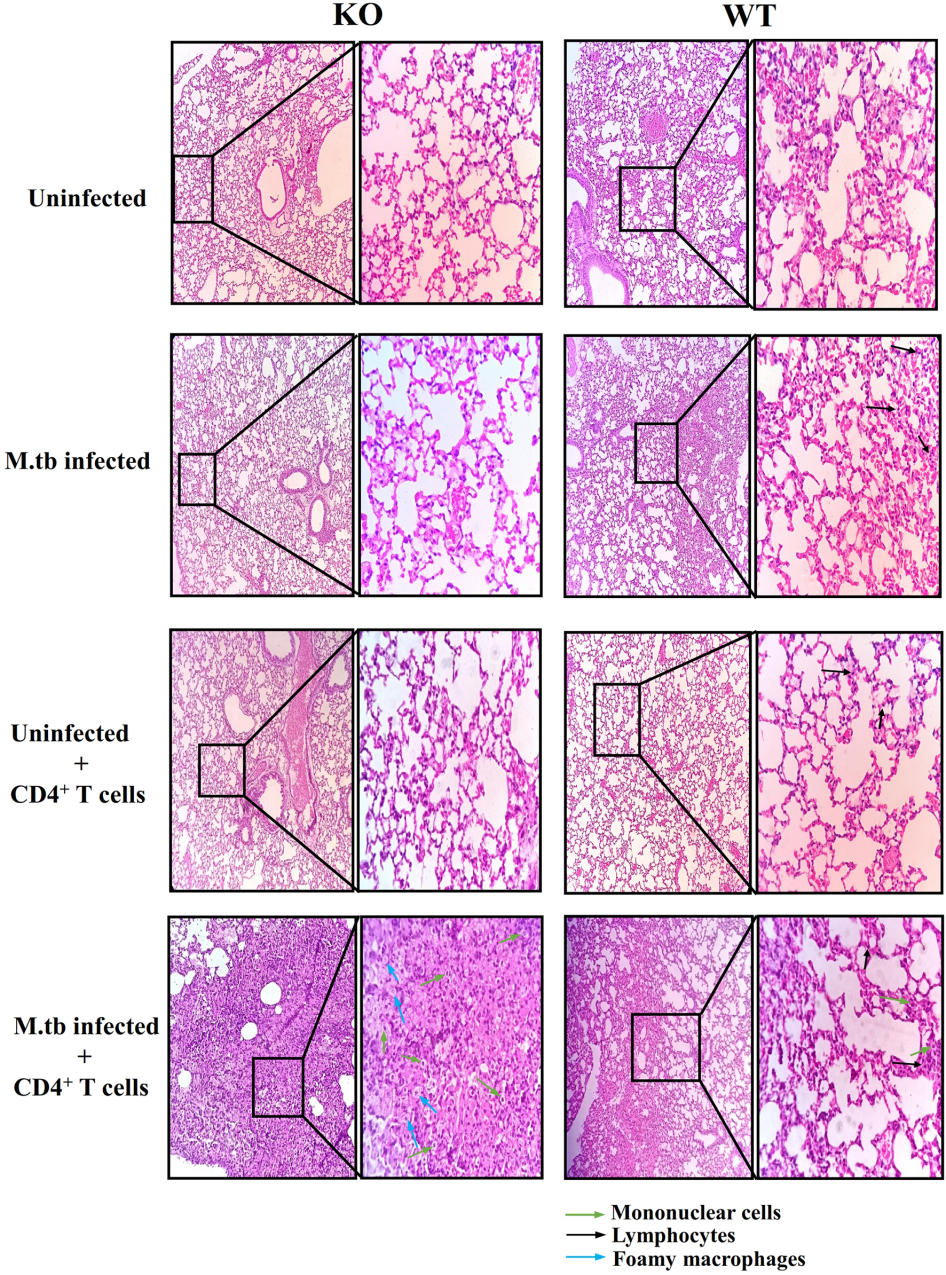
Lung tissue histopathology of TCRβ^−/−^ and WT mice after adoptive transfer of CD4+ T cells. Lung histopathology of uninfected, M.tb-infected TCRβ^−/−^, and WT mice before and after CD4+ T-cell transfer. The left panel shows KO mice lung sections, and the right one shows WT mice lung sections. Mononuclear cells are shown with green colored arrow and foamy macrophages are shown with blue colored arrows. Black colored arrow show lymphocytes.

Lung histopathology analysis was done in all the groups of TCRβ^−/−^ and WT mice (**Figure 3**). In M.tb-infected TCRβ^−/−^ mice in which CD4+ T cells were transferred, a large number of mononuclear cells can be seen in the lung sections as compared to WT mice (shown by green arrow). At higher magnification (100×), foamy macrophages (blue arrows) can be seen in the lung section of KO mice. No difference in the uninfected and only CD4+ T cell transferred group was observed between KO and WT mice. In the M.tb-infected WT mice, a higher number of mononuclear cells (mainly lymphocytes) were observed as compared to M.tb-infected TCRβ^−/−^ mice because there are no T cells in this group. These observations corroborate with the flow cytometry studies.

### Secretory Cytokines in the Macrophage Culture Supernatant

**Figure 4:**
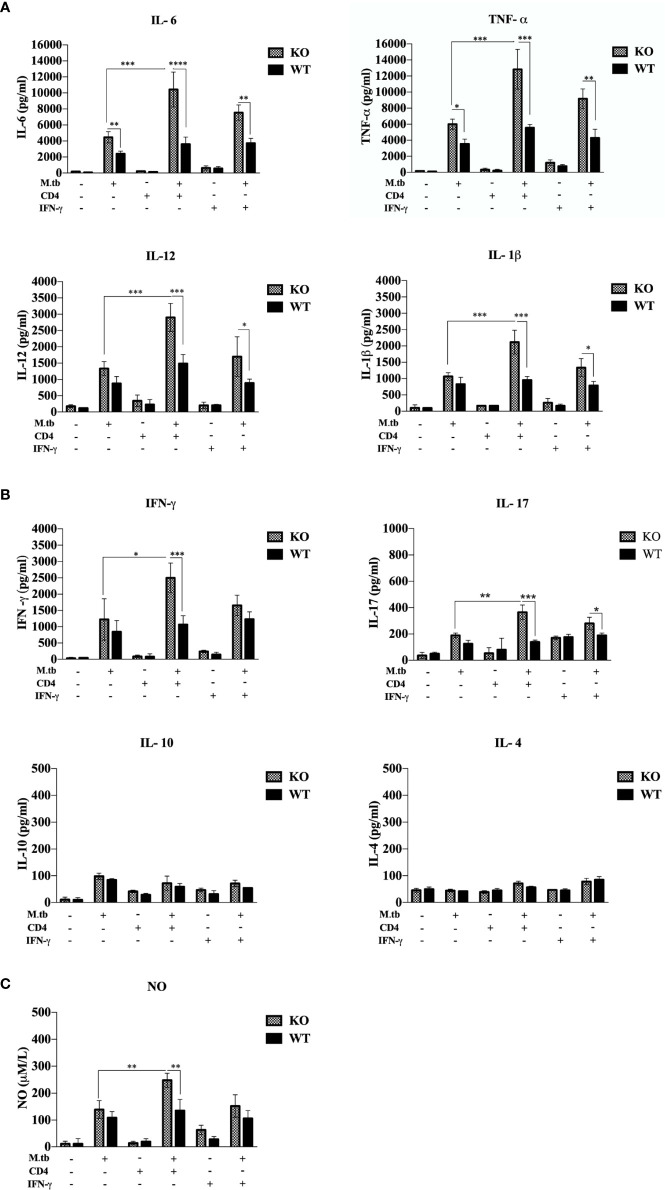
Secretory cytokines in the *ex vivo* M.tb-infected macrophages culture supernatant. TCRβ^−/−^ and WT mice peritoneal macrophages were infected with M.tb at MOI 10×. After 4 h of infection, CD4+ T cells or IFN-γ (20 IU/ml) were added to different groups. **(A)** Proinflammatory cytokine (IL-6, TNF-α, IL-12, and IL-1β) and **(B)** T-cell-secreted cytokine (IFN-γ, IL-4, IL-10, and IL-17) levels in culture supernatants were determined by CBA. **(C)** Level of nitric oxide in culture supernatant was checked indirectly by measuring nitrite concentration using Griess reagent. Statistical analysis was done by two-way ANOVA (Holm-Sidak method). Data are mean ± SEM of three independent experiments. *p < 0.02, **p <0.01, ***p < 0.0010, ****p < 0.00001.

Previous studies (19, 20) have shown that hyperactivated CD4+ T cells are responsible for pathogenesis of TB-IRIS, but how these CD4+ T cells become hyperactivated is not completely known. CD4+ T cells require help from antigen-presenting cells for their activation, and according to our hypothesis, these macrophages are somehow differently activated in the TCRβ^−/−^ mice. Hence, to examine the role of macrophages in the development of TB IRIS, their activation status was analyzed and compared between WT and TCRβ^−/−^ mice by measuring the levels of secretory cytokines in the culture supernatant of peritoneal macrophages. There were six groups in this experiment, i.e., only macrophages, macrophages infected with M.tb, macrophages co-cultured with CD4+ T cells, M.tb-infected macrophages co-cultured with CD4+ T cells, macrophages with IFN-γ alone, and M.tb-infected macrophages with IFN-γ. Higher levels of pro-inflammatory cytokines IL-6, TNF-α, IL-12, and IL-1β were detected in the culture supernatant of macrophages from TCRβ^−/−^ mice in which M.tb infection was given as compared to wild type. The levels of these cytokines further increased to a significant level after the addition of CD4+ T cells (**Figure 4A**). Another interesting observation was that in TCRβ^−/−^ mice macrophages, recombinant IFN-γ along with M.tb infection also increased the secretion of pro-inflammatory cytokines in culture supernatant, which is higher than M.tb alone but less than the M.tb+CD4+ T cell group (**Figure 4A**). Since CD4+ T cells were co-cultured with macrophages, we further checked the T-cell-secreted cytokines and found that level of Th1 (IFN-γ) and Th17 (IL-17) cytokines was high in the KO group while the level of Th2 (IL-4 and IL-10) cytokines was very low in both KO and WT groups (**Figure 4B**). Level of nitric oxide (NO), which plays a key role in the elimination of intracellular M.tb from infected macrophages, was also analyzed. Interestingly, significantly higher NO production by M.tb-infected TCRβ^−/−^ mouse macrophages was observed as compared to WT macrophages, which further increased after the addition of CD4+ T cells (**Figure 4C**). Previous studies (21) have shown that NO production depends on IFN-γ stimulation, but interestingly, here we found that TCRβ^−/−^ mouse macrophages produced copious amounts of NO even in the absence of IFN-γ.

### Expression of Activation Markers and Phago-Lysosomal Fusion Activity in *Ex Vivo* M.tb-Infected Macrophages

**Figure 5:**
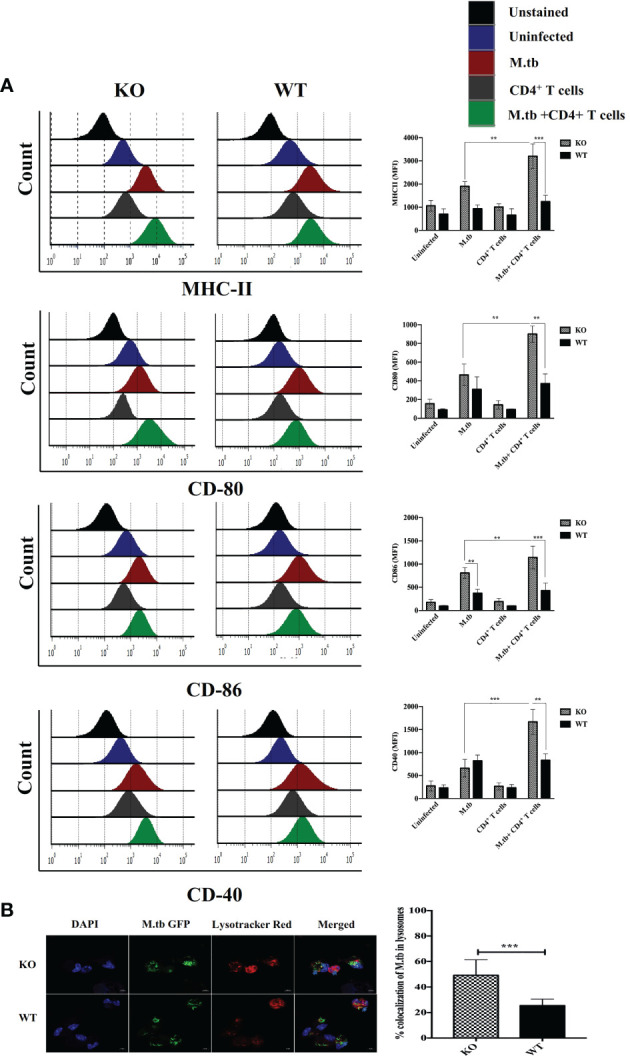
Expression of activation markers and phago-lysosomal fusion activity in *ex vivo* M.tb-infected macrophages. **(A)** TCRβ^−/−^ and WT mice peritoneal macrophages were infected with M.tb at MOI 10×. After 4 h of infection, CD4+ T cells were added in different groups, and expression of activation markers MHCII, CD80, CD86, and CD40 was analyzed by flow cytometry. **(B)** Macrophages were infected with M.tb-GFP at 10× MOI and percentage of M.tb in lysosomal compartment was analyzed by confocal microscopy (LSM 980), which reflects efficiency of phago-lysosomal fusion. Statistical analysis was done by two-way ANOVA (Holm-Sidak method). Data are mean ± SEM of three independent experiments.**p < 0.005, ***p < 0.0010.

To assess the *ex vivo* activation status of peritoneal macrophages, expression of MHCII, CD80, CD86, and CD40 was analyzed on the macrophages from TCRβ^−/−^and wild-type mice. There were four groups in this experiment, i.e., only macrophages, macrophages infected with M.tb, macrophages co-cultured with CD4+ T cells, and M.tb-infected macrophages co-cultured with CD4+ T cells (**Figure 5A**). After M.tb infection, the TCRβ^−/−^ mice macrophages showed significantly higher expression of MHCII and CD86 while expression of CD80 and CD40 was found to be similar in infected KO and WT mice macrophages, but interestingly, after the addition of CD4+ T cells, significantly higher expression of MHCII, CD80, CD86, and CD40 was observed in M.tb-infected KO mice macrophages as compared to WT macrophages. Co-stimulatory molecules (CD80/86) expressed by macrophages interact with CD28 on CD4+ T cells, which provide a second signal to CD4+ T cells for their activation and proliferation (22, 23). Significantly higher expression of co-stimulatory markers and MHCII provides evidence that macrophages from mice model of TB-IRIS are in more activated state as compared to their WT counterpart.

Phago-lysosomal trafficking is an important innate defense pathway that clears microbes by delivering them to lysosomes (24). Antigen presentation by MHCII requires processing of M.tb antigens in the lysosome. To examine the phago-lysosomal fusion status, co-localization of green fluorescent protein (GFP) expressing M.tb in lysosomes was checked in macrophages by using lysotracker red (**Figure 5B**). Lysotracker red is a specific dye that stains acidic compartments in the cells, so the GFP expressing bacteria that is inside the lysosome appears yellow. The twofold higher number of GFP expressing M.tb were found in the lysosomal compartment of TCRβ^−/−^ mice macrophages as compared to WT, showing that there is high phago-lysosomal fusion activity in TCRβ^−/−^ mice macrophages. These results suggested robust antigen presentation efficacy of TCRβ^−/−^ mice macrophages compared to wild-type macrophages. Furthermore, we got curious whether this higher inflammation affects the survival of macrophages. To investigate this, we examined the survival of KO and WT mice peritoneal macrophages after M.tb infection. For this, culture supernatants from M.tb-infected KO and WT mice, post 96 h of infection, were checked for lactate dehydrogenase secretion in *ex vivo* culture (LDH, which leaks out from dead cells). A low level of LDH activity in supernatants from M.tb-infected KO mice macrophages demonstrated a lower level of M.tb-induced cell death than WT macrophages (**Figure S3**). This higher viability of KO mice macrophages suggests that these macrophages can interact with CD4+ T cells for an extended period, potentially resulting in prolonged inflammation.

### Infiltration of Macrophages and Neutrophils in M.tb-Infected TCRβ^−/−^ and WT Mice Lungs Before and After CD4+ T Cell Transfer

We further analyzed the infiltration of macrophages in lungs of M.tb-infected KO mice and found that it was increased by about 15% as compared to uninfected mice. After adoptive transfer of CD4+ T cells, this was further increased by 50%–60% in infected KO mice while no such increase was observed in WT mice (**Figure S4**). When macrophage population was compared between infected KO *vs*. infected WT mice, 1.5-fold higher level of macrophages was observed in M.tb-infected KO lungs as compared to infected WT lungs. In addition, infiltration of neutrophils was analyzed. Neutrophil infiltration was found to be significantly high in M.tb-infected TCR β KO mice (40%–50% increase as compared to uninfected controls), which further increased by 50%–55% after transfer of CD4+ T cells. So, neutrophil infiltration was much higher than macrophages.

### Expression of Activation Markers on M.tb-infected TCRβ^−/−^ and WT Mice Lung Macrophages and Blood Monocytes 15 Days Post CD4+ T Cell Transfer

**Figure 6:**
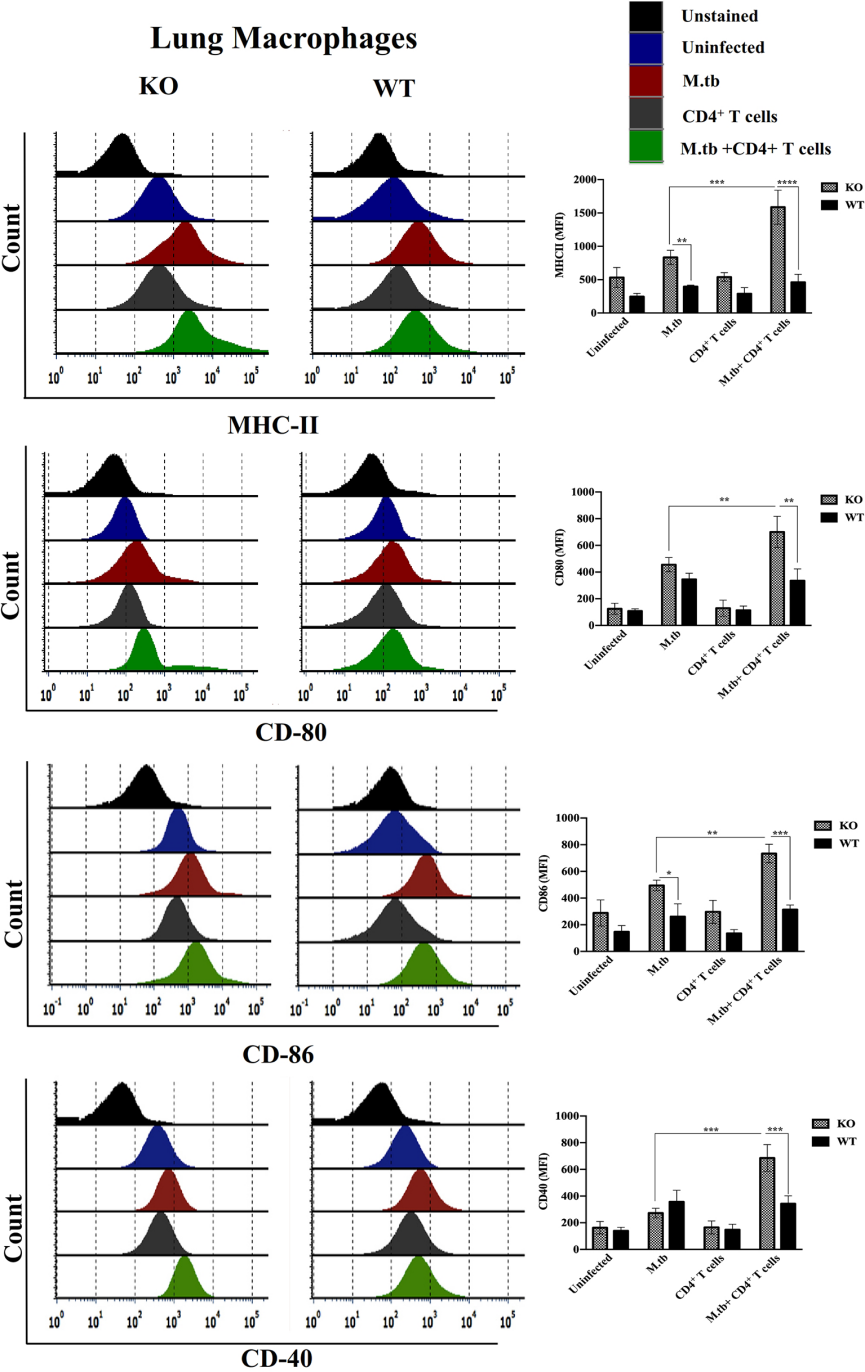
Expression of activation markers on M.tb-infected TCRβ^−/−^ and WT mice lung macrophages 15 days post CD4+ T-cell transfer. TCRβ^−/−^ and WT mice were challenged with M.tb (about 200 CFU) by aerosol route. After 30 days of infection, 2 × 10^6^ CD4+ T cells isolated from M.tb-infected WT CD45.1 C57BL/6 mice were adoptively transferred. Expression of MHCII, CD80, CD86, and CD40 on lung macrophages were analyzed by flow cytometry. Statistical analysis was done by two-way ANOVA (Holm-Sidak method). Data are mean ± SEM of three independent experiments. *p < 0.02, **p < 0.005, ***p < 0.0010, ****p < 0.00005.

**Figure 7:**
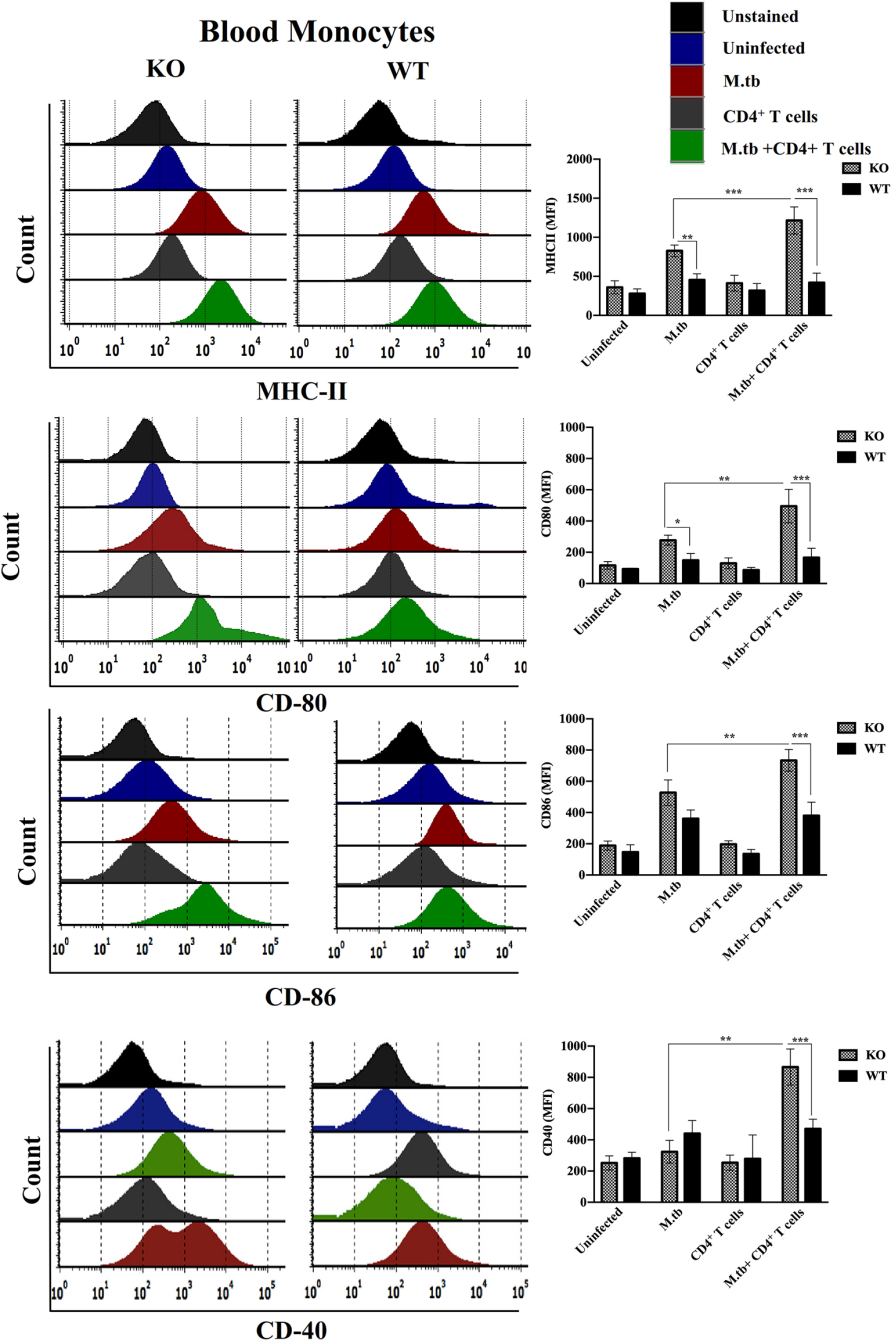
Expression of activation markers on M.tb-infected TCRβ^−/−^ and WT mice blood monocytes 15 days post CD4+ T-cell transfer. TCRβ^−/−^ and WT mice were challenged with M.tb (about 200 CFU) by aerosol route. After 30 days of infection, 2 × 10^6^ CD4+ T cells isolated from M.tb-infected WT CD45.1 C57BL/6 mice were adoptively transferred. Expression of MHCII, CD80, CD86, and CD40 on blood monocytes were analyzed by flow cytometry. Statistical analysis was done by two-way ANOVA (Holm-Sidak method). Data are mean ± SEM of three independent experiments. *p < 0.02, **p < 0.005, ***p < 0.0010.

Since we obtained interesting results in the *ex vivo* culture of peritoneal macrophages and found higher activation status of TCRβ^−/−^ mice macrophages, we further analyzed the activation status of lung and systemic macrophages in *in vivo* conditions. For this, mice were challenged with M.tb (about 200 CFU/mice) by aerosol route, and after 30 days of infection, CD4+ T cells were transferred from M.tb-infected WT mice to KO mice. There were four groups, i.e., uninfected control group, M.tb-infected group, only CD4+ T cells transferred group, and M.tb-infected group in which CD4+ T cells were transferred. After 15 days of CD4+ T-cell transfer, lungs from each group were collected and single-cell suspension was prepared and analyzed for the expression of macrophages activation markers. Similar to the *ex vivo* study, after M.tb infection, expression of MHCII and CD86 was higher in TCRβ^−/−^ mice (**Figure 6**). Lung macrophages from M.tb-infected TCRβ^−/−^ mice in which CD4+ T cells were adoptively transferred showed significantly higher expression of MHCII, CD80, CD86, and CD40 compared to WT. Since TB-IRIS is a systemic disease, expression of these activation markers was also checked and compared between blood monocytes of TCRβ^−/−^ and WT mice. Only M.tb infection resulted in the increased expression of MHCII and CD80 in TCRβ^−/−^ mice blood monocytes while similar to lung macrophages, adoptive transfer of CD4+ T cells in M.tb-infected TCRβ^−/−^ mice resulted in significantly higher expression of MHCII, CD80, CD86, and CD40 on blood monocytes compared to WT (**Figure 7**), which would make them potent cells to modulate the adaptive immune response. These results suggest that macrophages from TCR-β ^−/−^animals are robust and abnormally activated, which supports our hypothesis.

### Serum Cytokine Level in M.tb-Infected TCRβ^−/−^ and WT Mice 15 Days Post CD4+ T Cell Transfer

**Figure 8:**
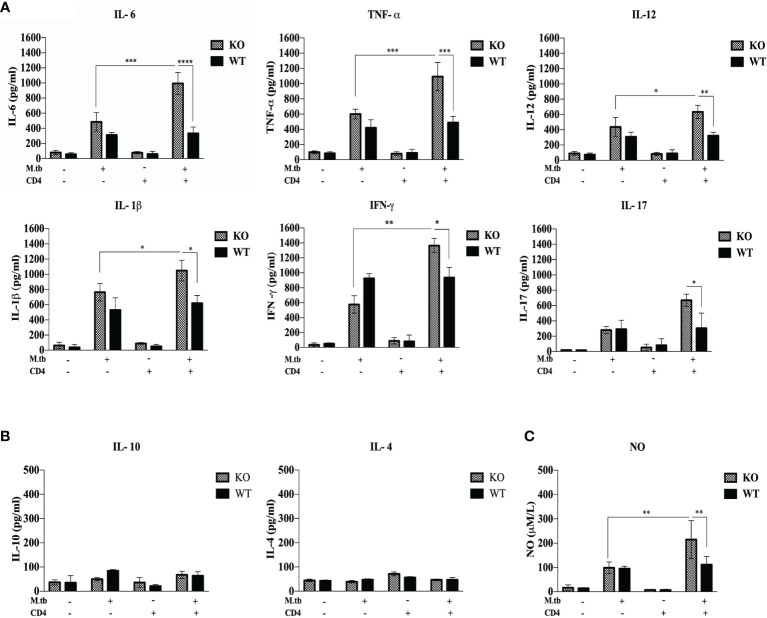
Serum cytokine level in M.tb-infected TCRβ^−/−^ and WT mice 15 days post CD4+ T-cell transfer. TCRβ^−/−^ and WT mice were challenged with M.tb (about 200 CFU) by aerosol route. After 30 days of infection, 2 × 10^6^ CD4+ T cells isolated from M.tb-infected WT CD45.1, C57BL/6 mice were adoptively transferred in one of the M.tb-infected groups. Serum was isolated 15 days post adoptive CD4+ T-cell transfer, and **(A)** levels of proinflammatory cytokines (IL-6, TNF-α, IL-12, IFN-γ, IL-17, and IL-1β) and **(B)** anti-inflammatory cytokines (IL-4 and IL-10) were measured by CBA. **(C)** Level of nitric oxide was checked indirectly by measuring nitrite concentration using Griess reagent. Statistical analysis was done by two-way ANOVA (Holm-Sidak method). *p < 0.02, **p < 0.005, ***p < 0.0010, ****p < 0.00001.

Furthermore, to study the systemic response, serum cytokines in M.tb-infected TCRβ^−/−^ and WT mice were checked. After M.tb infection, a higher level of IL-6, TNF-α, IL-12, and IL-1β was observed in TCRβ^−/−^ mice, as compared to WT mice, which further increased significantly when CD4+ T cells were transferred (**Figure 8A**). When compared to WT, M.tb-infected TCRβ^−/−^ mouse serum showed substantially more NO level, which increased further when CD4+ T cells were introduced in M.tb-infected TCRβ^−/−^ mice (**Figure 8C**). We also checked the level of anti-inflammatory cytokines in serum; a low level of IL-4 and IL-10 was observed in both TCRβ^−/−^ and WT mice serum (**Figure 8B**). Pro-inflammatory cytokines are in charge of launching a strong response against exogenous pathogens while anti-inflammatory cytokines are important for reducing the inflammatory response and preserving homeostasis for proper organ function. The excessive output of these mediators could be playing crucial role in the development of IRIS syndrome.

### mRNA Expression of Inflammation Associated Markers in Lung Macrophages

As a critical component of innate immunity, macrophages serve as the first-line defense of M.tb infection (25). To further explore the effect of CD4+ T cell deficiency and M.tb infection on macrophages, lung macrophages were isolated from different groups of M.tb-infected and uninfected, TCRβ^−/−^and WT animals post 15 days of CD4+ T cell transfer. mRNA expression of various inflammation-associated markers like chemokines, matrix metalloproteases, M.tb-associated pattern recognition molecules, and other inflammation regulatory molecules was analyzed.

**Figure 9:**
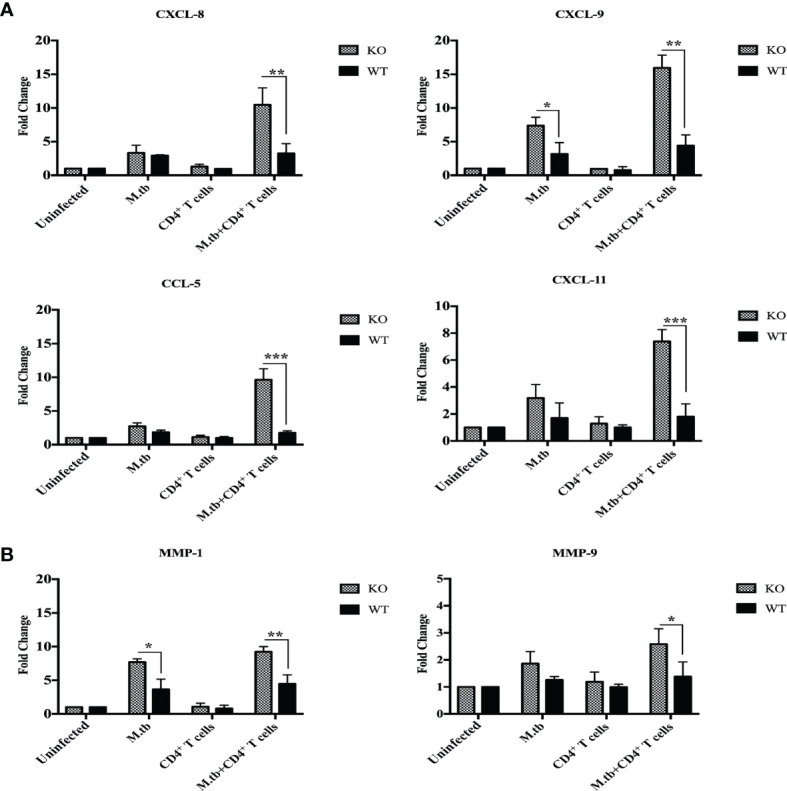
mRNA expression of chemokines and MMPs in lung macrophages. mRNA expression of secretory chemokines and MMPs was analyzed in macrophages from lungs of M.tb-infected or uninfected TCRβ^−/−^ and WT mice 15 days post CD4+ T-cell transfer. Level of chemokines **(A)** CXCL8, CXCL9, and CXCL11 CCL5 and **(B)** MMP-1 and MMP-9 were compared by qRT-PCR. Data are mean ± SEM of three independent sets of experiments. Analysis was done by two-way ANOVA (Holm-Sidak method). Data are mean ± SEM of three independent experiments. *p < 0.02, **p < 0.005, ***p < 0.0010.

Chemokines are chemical messengers secreted by immune and non-immune cells that play a critical role in recruiting other immune cells to the infection site (26). In lung macrophages, mRNA expression of chemokines CCL2, CCL3, CCL5, CXCL1, CXCL2, CXCL8, CXCL9, and CXCL11 and chemokine receptors CCR1 and CCR2 was compared between different groups of TCRβ^−/−^ and wild-type mice by qRT-PCR. In only M.tb-infected group, mRNA expression of CXCL8, CXCL11, and CCL5 was approximately threefold high in TCRβ^−/−^ and twofold high in wild type as compared to uninfected control mice (**Figure 9A**). Interestingly, after adoptive transfer of CD4+ T cells in M.tb-infected groups, mRNA expression of CXCL11 and CCL5 increased about 8-fold, and CXCL8 increased about 10-fold in TCRβ^−/−^ mice, while no change was observed in wild-type mice (**Figure 9A**). Similarly, mRNA expression of CXCL9 increased about 5-fold in only M.tb-infected TCRβ^−/−^ mice group and about 15-fold in M.tb-infected TCRβ^−/−^ mice group in which CD4+ T cells were transplanted (**Figure 9A**). In contrast, about twofold rise was seen in the wild-type group in both M.tb-infected and M.tb+ CD4+ T-cell-transferred groups. After M.tb infection, mRNA expression of CCR2 increased about 5-fold in M.tb-infected TCRβ^−/−^ mice compared to 1.5-fold increase in WT mice whereas CD4+ T cell transfer resulted in an approximately 8-fold rise in KO mice, but no such increase was found in WT. No difference in the mRNA expression of CCR1 was observed between different groups of KO and WT mice (**Figure S5**). mRNA expression of other chemokines, CCL2, CCL3, CXCL1, and CXCL2, was also analyzed but no significant change was detected (data not shown).

Matrix metalloproteinases (MMPs) are a group of proteases that destroy extracellular matrix components and are believed to play a role in tuberculosis-related lung injury (27, 28). During TB infection, MMPs may facilitate various stages of lung remodeling (29). MMP1, MMP3, MMP8, MMP9, and MMP11 mRNA expression in lung macrophages have been examined before and after M.tb infection and CD4+ T cell transfer. mRNA expression of MMP1 was about 6-fold higher in M.tb-infected TCRβ^−/−^ mice lung macrophages, which increased to about 10-fold upon CD4+ T-cell transfer, compared to the 2-fold increase in M.tb + CD4 T-cell-transferred group of WT mice (**Figure 9B**). No change was observed in mRNA expression of MMP9 in TCRβ^−/−^ and wild-type mice after M.tb infection, but after CD4+ T-cell transfer, mRNA expression of MMP9 increased to about 1.5 times in M.tb-infected KO mice. These results show higher expression of CXCL8, CXCL9, CXCL11, CCL5, MMP1, and MMP9 in the mouse model of TB-IRIS, which could be possibly involved in the pathogenesis of IRIS disease.

**Figure 10:**
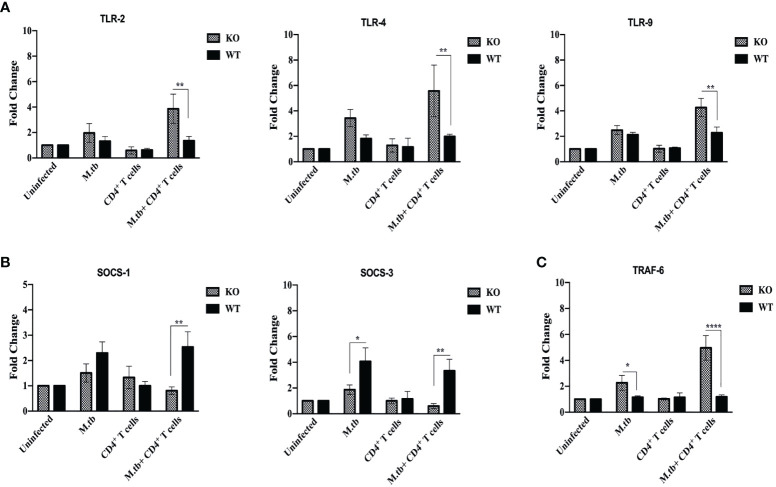
mRNA expression of TLRs, SOCS, and TRAF-6 in lung macrophages. mRNA expression of **(A)** TLR2, TLR4, and TLR9, **(B)** SOCS1 and SOCS3, and **(C)** TRAF-6 was determined in macrophages from lungs of M.tb-infected or uninfected TCRβ^−/−^and WT mice 15 days post CD4+ T-cell transfer. Data are mean ± SEM of three independent sets of experiments. Analysis was done by two-way ANOVA (Holm-Sidak method). *p < 0.02, **p < 0.005, ****p < 0.00005.

Pattern recognition molecules like TLRs and feedback inhibition regulating molecules like Suppressor of Cytokines Signaling (SOCS) were also analyzed and compared between TCRβ^−/−^ and wild-type mice lung macrophages. Toll-like receptors are very crucial pattern recognition receptors in the recognition of M.tb. Recognition of microbial products by these receptors trigger functional maturation and initiation of antigen-specific adaptive immune responses (30). So, we further analyzed whether these are differentially expressed in TCRβ^−/−^ *vs*. WT mice. mRNA expression of TLR2, TLR4, and TLR9 were found to be significantly increased in lung macrophages of M.tb-infected TCRβ^−/−^ mice as compared to WT (**Figure 10A**).

The SOCS proteins function as feedback inhibitors of the various signaling pathways and can terminate innate and adaptive immune responses and thus control inflammation in a normal situation (31). So, the expression of SOCS1 and SOCS3 in lung macrophages was analyzed. Interestingly, mRNA expression of SOCS1 and SOCS3 was found to be low in infected lung macrophages of TCRβ^−/−^ mice as compared to WT lung macrophages (**Figure 10B**).

Tumor necrosis factor receptor-associated factor 6 (TRAF6), an E3 ubiquitin ligase, is a signaling protein of the interleukin-1 receptor (IL-1R)/Toll-like receptor (TLR) family and the TNFR superfamily (32). In this study, we also examined the mRNA expression of TRAF-6 in lung macrophages. Following M.tb infection, it was observed that TRAF-6 mRNA expression in TCRβ^−/−^ and WT mice lung macrophages increased by 2.5-fold when compared to uninfected control, and this further increased to 5-fold after CD4+ T cell transfer in TCRβ^−/−^ mice (**Figure 10C**). After M.tb infection and CD4+ T-cell transfer, no statistically significant change in the mRNA expression of TRAF-6 was detected in wild-type lung macrophages.

## Discussion

TB-IRIS is the most challenging aspect of scaling up ART in HIV-M.tb coinfected patients as it results in an aberrant inflammatory response in a subset of co-infected patients. Both terminally differentiated and effector memory Th1-type mycobacteria-specific CD4+ T cells that expand in number with ART show excessive release of IFN-γ and other proinflammatory cytokines. Pathology of IRIS has been reported to be associated with adaptive immune factors, but a couple of studies suggest a probable role of macrophages and monocytes (14, 16, 33). Activation of CD4+ T cells requires help from APCs: antigen presentation with MHCII, secreted cytokines, and other cell surface molecular interactions. In case of tuberculosis, macrophages are the major host cells exploited by M.tb for their growth and duplication; hence, it was hypothesized that they would play an important role in the development of pathogenesis of TB-IRIS. This study is an attempt to examine in detail the role of macrophages in an animal model of TB-IRIS as only a few reports have suggested that dysregulated monocyte function (analyzed in patient’s blood) could be responsible for this syndrome in patients where soluble markers for inflammation were reported to be increased (15, 16, 34).

In this study, the effect of CD4+ T cell deficiency on macrophage function and the underlying mechanisms in the context of M.tb infection have been analyzed. Here, we have described an animal model of experimental TB-IRIS, i.e., TCRβ^−/−^ mice that lack all the T cells with α-β T cell receptors and represent a similar condition like AIDS patient (in which, however, CD8+ T cells are present), and transfer of CD4+ T cells creates a similar condition that occurs after ART. Transfer of CD4+ T cells from M.tb-infected C57Bl/6 mice into TCRβ^−/−^ mice during chronic mycobacterial infection resulted in fatal inflammatory disease, where all the mice succumbed by day 25. Body temperature, which is one of the hallmarks of inflammation, was also checked. It was observed that after M.tb infection, there was an increase in the temperature in both TCRβ^−/−^ and wild-type mice, but after CD4+ T cell transfer, interestingly there was a decrease in core and surface body temperature of TCRβ^−/−^ mice, while there was no such change in the body temperature of wild-type mice. Fever is the most frequent thermal manifestation in infections of different severities ranging from minor viral illnesses to bacterial sepsis, while hypothermia characteristically develops in severe cases of inflammation. Fever and hypothermia correspond to mild and severe forms of systemic inflammation, respectively (35, 36). Hypothermia instead of fever observed in M.tb-infected KO mice after CD4+ T cell transfer could be due to excessive inflammation (17).

When comparing the pulmonary and extrapulmonary bacterial burden in TCRβ^−/−^ and C57Bl/6 WT mice before and after CD4+ T cell transfer, it was observed that in contrast to WT mice, TCRβ^−/−^ mice had about one log higher bacillary load in the lung, spleen, and liver at day 30 after infection, which could be due to the absence of T-cell response, which otherwise is essential for effective elimination of M.tb from the host. Interestingly, after the adoptive transfer of CD4+ T cells in TCRβ^−/−^ mice, the bacterial load decreased significantly in all the organs studied. Since the number of T cells transferred was not high enough to reduce the bacterial load by cytotoxic activity or through other protective mechanisms, this might be because of hyperimmune response that reduced the bacterial burden significantly. As KO mice had higher bacterial load before transfer of CD4+ T cells, this could also be one of the factors responsible for the development of TB-IRIS symptoms as this is generally accepted that high mycobacterial load prior to commencing ART is a risk factor for TB-IRIS (37).

Histopathological analysis of the lung section showed that there was no difference in the uninfected TCRβ^−/−^ and wild-type lungs while a high number of lymphocytes were observed after M.tb infection, in the lungs of WT mice, which was expected as KO mice lack T cells. Infiltration of mononuclear cells in the lung sections of TCRβ^−/−^ mice was substantially high after adoptive transfer of CD4+ T cells. These observations corroborate with flow cytometry analysis where significantly higher infiltration of macrophages was seen in the lungs of M.tb-infected TCRβ^−/−^ mice after CD4+ T cell transfer. Excessive infiltration of mononuclear cells could be responsible for higher proinflammatory cytokines and chemokines observed in this animal model of TB-IRIS.

In the current study, both *ex vivo* and *in vivo* analysis of macrophages provides evidence that macrophages from TCRβ^−/−^ mice are hyperactivated, secrete higher amount of proinflammatory cytokines, and express higher level of cell surface activation markers and MHCII as compared to WT mice. To check whether this significant increase in proinflammatory cytokines after the addition of CD4+ T cells is because of only IFN-γ from CD4+ T cells or some other interaction is also required, one additional group was taken, where recombinant IFN-γ was added. Although the addition of IFN-γ increased the levels of pro-inflammatory cytokines in KO macrophages culture supernatant, it was substantially lower as compared to the CD4+ T cell group, which suggest that the cross-talk or interaction between T cells and macrophages is crucial for pathogenesis of TB-IRIS. Cytokines are known to be protagonists in the pathology of number of diseases including IRIS. High secretion of proinflammatory cytokines by M.tb-infected macrophages could be responsible for dysregulated inflammation in the TCRβ^−/−^ mice, which lead to detrimental lung pathology. Among the proinflammatory cytokines found in large amount in the IRIS model, IL-6 is a pleiotropic cytokine that plays an important role in the initiation of T cell activation (38) and proliferation by suppressing regulatory T cells (39). Another important proinflammatory cytokine that was found very high in KO macrophage culture supernatant was IL-1β, which causes infiltration of neutrophils at the site of infection and has complex cross-talk with other mediators (40), which is in keeping with high infiltration of neutrophils and Th17 response observed in the mouse model of TB-IRIS. Exposure of IL-12 and IFN-γ greatly enhances the macrophage function in terms of antigen processing and presentation as observed in this study as well (41). Another cytokine expressed at a high level, TNF-α is one of the main cytokines for septic shock and induces vasodilation, which promotes infiltration of lymphocytes and neutrophils at the site of inflammation (42).

Macrophages from the mouse model of TB-IRIS showed upregulated expression of MHCII, CD80, CD86, and CD40. The costimulatory molecules (CD80 and CD86) play an important role in T-cell activation, and their interaction with CD28 on T cells enhances the production of IL-1, IL-6, and TNF-α, which corroborates our findings and supports our hypothesis (43). Similarly, the interaction of CD40 on macrophages with CD40L on T cells stimulates the production of IL-12 by macrophages (44). IL-17 response to M.tb also requires CD40-dependent co-stimulation (45). These pro-inflammatory cytokines seem to be the protagonists in the pathology of IRIS. To ascertain whether this abnormal behavior of macrophages from TCRβ^−/−^ mice is because of T cell deficiency or if any developmental defect was present, we had analyzed the expression level of activation markers along with cytokine secretion from bone marrow-derived macrophages and interestingly found no such difference (data not shown), suggesting that abnormal hyperactivation of macrophages could be because of T-cell deficiency.

The key function of macrophage innate response against pathogens is to kill the phagocytosed bacteria by delivering it to the acidic lysosomal compartment. Hence, we compared the phago-lysosomal fusion activity between KO and WT mice macrophages. TCRβ^−/−^ mice macrophages showed enhanced phagosome maturation and fusion with the lysosome, suggesting higher antigen processing and presentation efficacy compared to WT macrophages. Interestingly, these macrophages also had higher viability as compared to WT, which would help these macrophages to interact with CD4+ T cells for a longer duration of time that could result in prolonged inflammation.

After getting these interesting results in an *ex vivo* study of macrophages, *in vivo* activation status of lung macrophages was analyzed before and after the adoptive transfer of CD4+ T cells. Here, similar to an *ex vivo* study, adoptive transfer of CD4+ T cells in M.tb-infected mice resulted in upregulated expression of MHCII, CD80, CD86, and CD40 while no such change was observed in WT macrophages. Since TB-IRIS is a systemic disease, we further checked the activation status of blood monocytes along with serum cytokines. Similar to lung macrophages, blood monocytes from M.tb-infected KO mice showed increased expression of MHCII and CD80 and adoptive transfer of CD4+ T cells resulted in further increase in expression of activation markers. Consistent with previous reports, we confirmed that after transfer (reconstitution) of CD4+ T cells, serum level of pro-inflammatory cytokines was found to be also high in the mouse model of TB-IRIS.

To better understand the immune pathways that result in the development of TB-IRIS, further studies were done. In this mouse model of TB-IRIS, higher expression of TLR-2, TLR-4, and TLR-9 was observed in lung macrophages, suggesting that these macrophages are more efficient in recognizing the M.tb, besides secretion of higher amount of proinflammatory cytokines. These macrophages also secreted higher amount of inflammation-associated chemokines, which are responsible for recruiting pro-inflammatory cells at the site of infection, and MMPs, which are responsible for tissue damage. mRNA expression of CXCL8, CXCL9, CXCL11, and CCL5 was found to be high in lung macrophages of TCRβ^−/−^ mice, which significantly increased after adoptive transfer of CD4+ T cells. Among these chemokines, CXCL8 is a potent chemoattractant for neutrophils, which causes degradation and morphological changes in the target cells (46) and is integral in inflammatory diseases. Similarly, CXCL9 is a strong T-cell chemoattractant at the site of inflammation and also promotes differentiation and multiplication of leukocytes (47). CXCL11 is similar to CXCL8 and CXCL9 in function, but it is less potent. CCL5 is a chemoattractant for T cells, eosinophils, and basophils, and it is involved in leukocyte recruitment to the inflammatory site (48).

MMPs are believed to play an important role in tuberculosis-related lung injury. mRNA expression of MMP1 and MMP9 was found to be high in lung macrophages of M.tb-infected TCRβ^−/−^ mice post 15 days of CD4+ T cell transfer as compared to WT. MMP1 is the main chemokine responsible for alveolar wall damage and matrix destruction, secreted by monocytes and macrophages (49). Similarly, MMP9 is also responsible for ECM degradation and activation of IL-1β (50). Our results are consistent with MMP1 and MMP9’s tissue-destructive function. The upregulation of chemokines suggests a role for both T cells and macrophages emphasizing the interaction of innate and adaptive immune cells in the pathogenesis of IRIS.

The SOCS proteins function as feedback inhibitors of the many inflammatory signaling pathways and can downregulate innate and adaptive immune responses and thus control inflammation in a normal situation (31). Interestingly, mRNA expression of SOCS1 and SOCS3 were found to be low in infected lung macrophages of TCRβ^−/−^ mice as compared to WT macrophages. Tumor necrosis factor receptor-associated factor 6 (TRAF6) is a signaling protein of the interleukin-1 receptor (IL-1R)/Toll-like receptor (TLR) family and the TNFR superfamily. TRAF6 inhibition has previously been demonstrated to decrease cytokines, attenuate immunological responses, and promote M.tb survival (32). It was observed that M.tb infection resulted in increased expression of TRAF6 in TCRβ−/− as well as in wild-type mice but transfer of CD4+ T cells further significantly increased the mRNA expression of TRAF6 in TCRβ^−/−^ mice while no change was found in wild-type mice. These findings provide evidence that in the absence of T cells, there is inadequate control of inflammatory activation of macrophages due to dysregulated feedback mechanisms; hence, after interaction with reconstituted CD4+ T cells, this leads to the cytokine storm orchestrated by hyperactivated macrophages with higher secretion of MMPs and chemokines resulting in tissue damage and disease pathology.

Findings of this study suggest that M.tb-associated IRIS originates from hyperactive myeloid cells in terms of expression of activation markers/pathogen recognition receptors/antigen processing and presentation, induction of proinflammatory cytokines, and dysregulated control of inflammatory pathways, which, in turn, lead to inflammatory syndrome after interaction with reconstituted CD4+ T cells.

There are certain limitations of the model described, in its reflection of the physiology of IRIS in AIDS patients. AIDS patients have very low level of circulating CD4+ T cells, while in our animal model, TCRβ^−/−^ mice completely lack all the T cells. Also, being a mouse model, it does not show the full spectrum of the disease manifestation as in a patient. Although the model described in this work does not represent some of the aspects of IRIS in AIDS patients, insights into the pathogenesis of IRIS disease from our study have important implication for the understanding of M.tb-associated IRIS. A better understanding of the underlying mechanisms in the pathology of TB-IRIS will help to identify potential biomarkers for disease development in a subset of patients and would help in designing therapeutic strategies for this syndrome.

## Conclusions

This study aims to fill the gaps in the understanding of mechanisms leading to development of TB-IRIS. Previous studies have shown that hyperactive CD4+ T cells are responsible for disease induction and our study adds to this understanding by highlighting that macrophages in the absence of T cells are in a higher activated state, which results in hyperactivation of reconstituted CD4+ T cells and contribute to the pathology of TB-IRIS. The study provides evidence that M.tb-infected macrophages from the mouse model of experimental IRIS express higher cell surface activation markers even before reconstitution of CD4+ T cells. These parameters could be used in the development of biomarkers for the prognosis of TB-IRIS before the start of ART in M.tb-HIV co-infected patients.

## Data Availability Statement

The original contributions presented in the study are included in the article/**Supplementary Material**. Further inquiries can be directed to the corresponding author.

## Ethics Statement

The animal study was reviewed and approved by National Institute of Immunology’s Institutional Animal Ethics Committee Serial number IAEC#443/17.

## Author Contributions

LP, PK, and SB contributed to study design, data analysis, and data interpretation. LP, RN, GR, and BS have performed the experiments. LP and GR have contributed to manuscript preparation. LP and SB have read and approved the final manuscript. All authors contributed to the article and approved the submitted version.

## Funding

This work was supported by a grant from the Department of Biotechnology, Government of India (102/IFD/SAN/3646/2017-2018) and a core research grant of the National Institute of Immunology, New Delhi, India.

## Conflict of Interest

The authors declare that the research was conducted in the absence of any commercial or financial relationships that could be construed as a potential conflict of interest.

## Publisher’s Note

All claims expressed in this article are solely those of the authors and do not necessarily represent those of their affiliated organizations, or those of the publisher, the editors and the reviewers. Any product that may be evaluated in this article, or claim that may be made by its manufacturer, is not guaranteed or endorsed by the publisher.

## Supplementary Material

Supplementary Figure 1 Purity assessment and CFSE labelling of CD4+T cells after MACS sorting before adoptive transfer. Percentage purity of MACS sorted CD4+ T-cells was evaluated by FACS analysis based on surface markers. 90 to 95 percent pure cells population was used for adoptive transfer. Before transfer, cell were stained with CFSE for analysis of *in vivo* proliferation.

Supplementary Figure 2 Proliferation of adoptively transfered CD4+T cells in M.tb infected TCRβ^−/−^ and WT mice. Purified, CFSE labelled CD4+ T cells were transferred by i.v. route in M.tb infected KO/WT mice. After 72 hours of transfer, the CFSE dilution of CD4+ T cells in M.tb infected KO and WT mice blood was compared.

Supplementary Figure 3 Viability of KO macrophages after 7 days of M.tb infection. Lactate dehydrogenase (LDH) assay was performed and percentage of viable macrophages were calculated. Data are mean ± SEM of three independent experiments. **p < 0.005.

Supplementary Figure 4 Infiltration of macrophages and neutrophils in M.tb infected TCRβ^−/−^ and WT mice lungs. Single cell suspension was prepared from lungs of different groups of KO and WT mice. Cells were stained with anti-CD11b, anti-F4/80 and anti-Gr1 fluorescent antibodies to analyze macrophages and neutrophils populations by flow cytometry. Representative flow cytometry plots of are presented, **(A)** M.tb infected KO mice lung single cell suspension after CD4+ T cells transfer, **(B)** M.tb infected WT mice lung single cell suspension after CD4+ T cells transfer are presented. Macrophages are F4/80^+^ and CD11b^+^ double positive. Neutrophils are Gr1^+^and CD11b^+^double positive. **(C)** Percentage population of macrophages and neutrophils in lungs of different groups of KO and WT mice are shown.

Supplementary Figure 5 mRNA expression of chemokine receptors in lung macrophages. mRNA expression of chemokine receptors was evaluated in macrophages from lungs of M.tb infected and uninfected TCRβ^−/−^ and WT mice 15days post CD4+ T cells transfer. Data are mean ± SEM of three independent sets of experiments. Analysis was done using two-way ANOVA. **p < 0.005, ****p<0.00005.

Supplementary Figure 6 Purity analysis of lung macrophages for mRNA isolation. Single cell suspension was prepared from lungs and allowed to adhere in culture flask for 5 hours; non-adherent cells were removed by washing twice with PBS. Purity of these cells were determined by flow cytometry. Cells were stained with anti-CD11b, anti-F4/80 and anti- CD14 fluorescent antibodies to analyze macrophages population.
