# Supplementary figures and images for: Macrophages Are the Key Players in Promoting Hyper-Inflammatory Response in a Mouse Model of TB-IRIS
Source: Front Immunol. 2021 Nov 26;12:775177. doi: 10.3389/fimmu.2021.775177 (PMC8662811; doi:10.3389/fimmu.2021.775177)

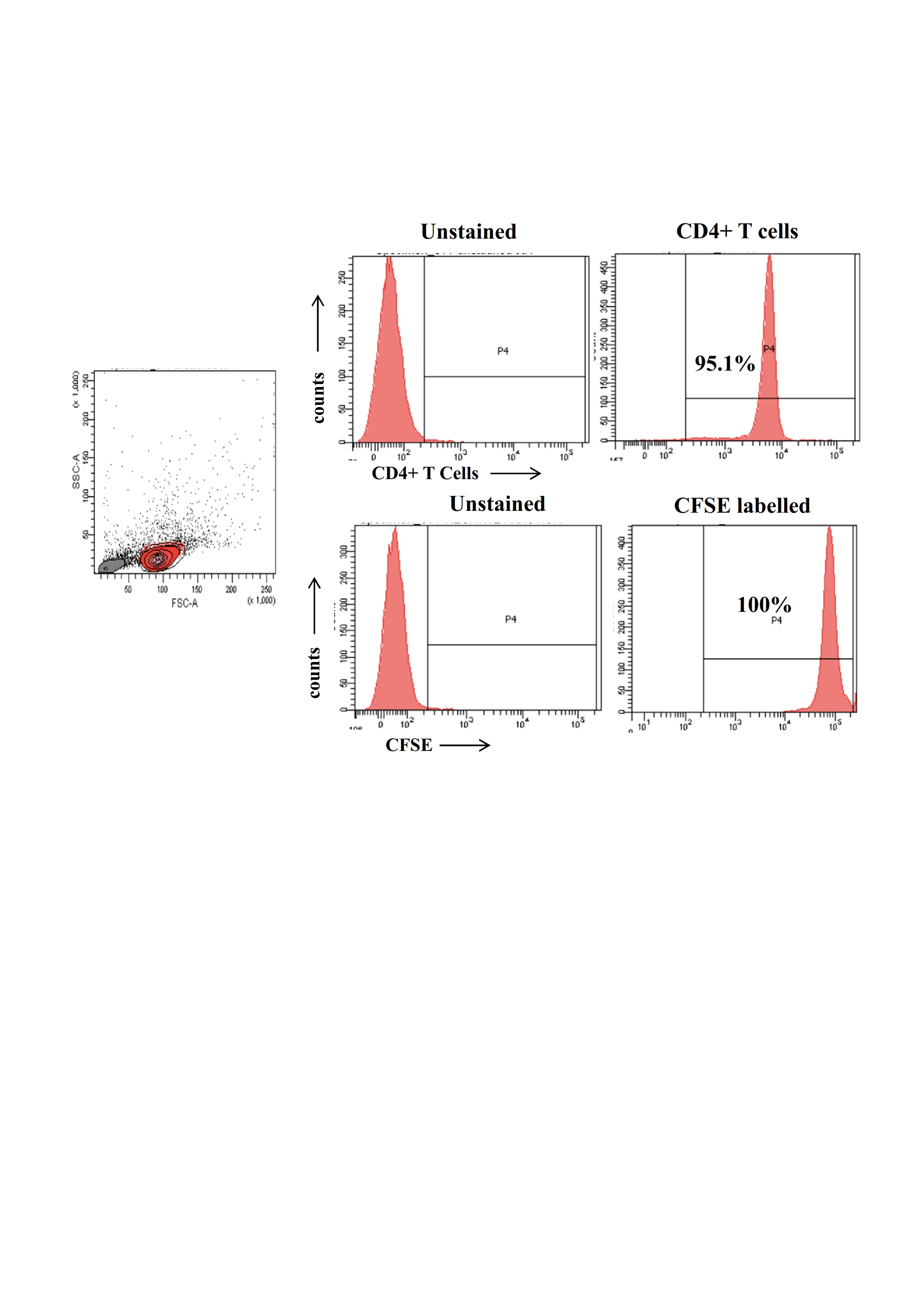

Supplement: Supplementary Figure 1 — Purity assessment and CFSE labelling of CD4+T cells after MACS sorting before adoptive transfer. Percentage purity of MACS sorted CD4+ T-cells was evaluated by FACS analysis based on surface markers. 90 to 95 percent pure cells population was used for adoptive transfer. Before transfer, cell were stained with CFSE for analysis of in vivo proliferation. [file Image_1.jpeg]

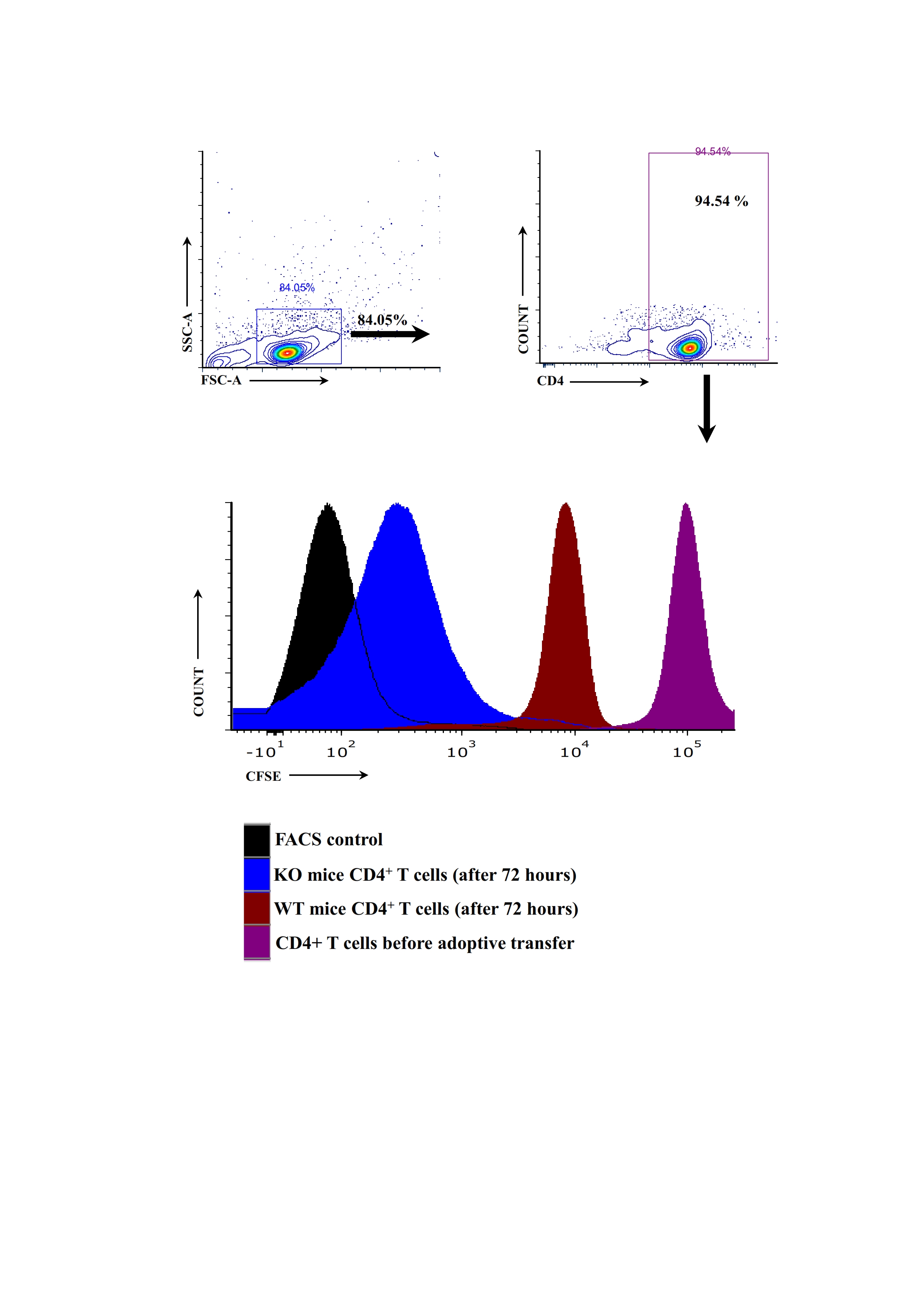

Supplement: Supplementary Figure 2 — Proliferation of adoptively transfered CD4+T cells in M.tb infected TCRβ−/− and WT mice. Purified, CFSE labelled CD4+ T cells were transferred by i.v. route in M.tb infected KO/WT mice. After 72 hours of transfer, the CFSE dilution of CD4+ T cells in M.tb infected KO and WT mice blood was compared. [file Image_2.jpeg]

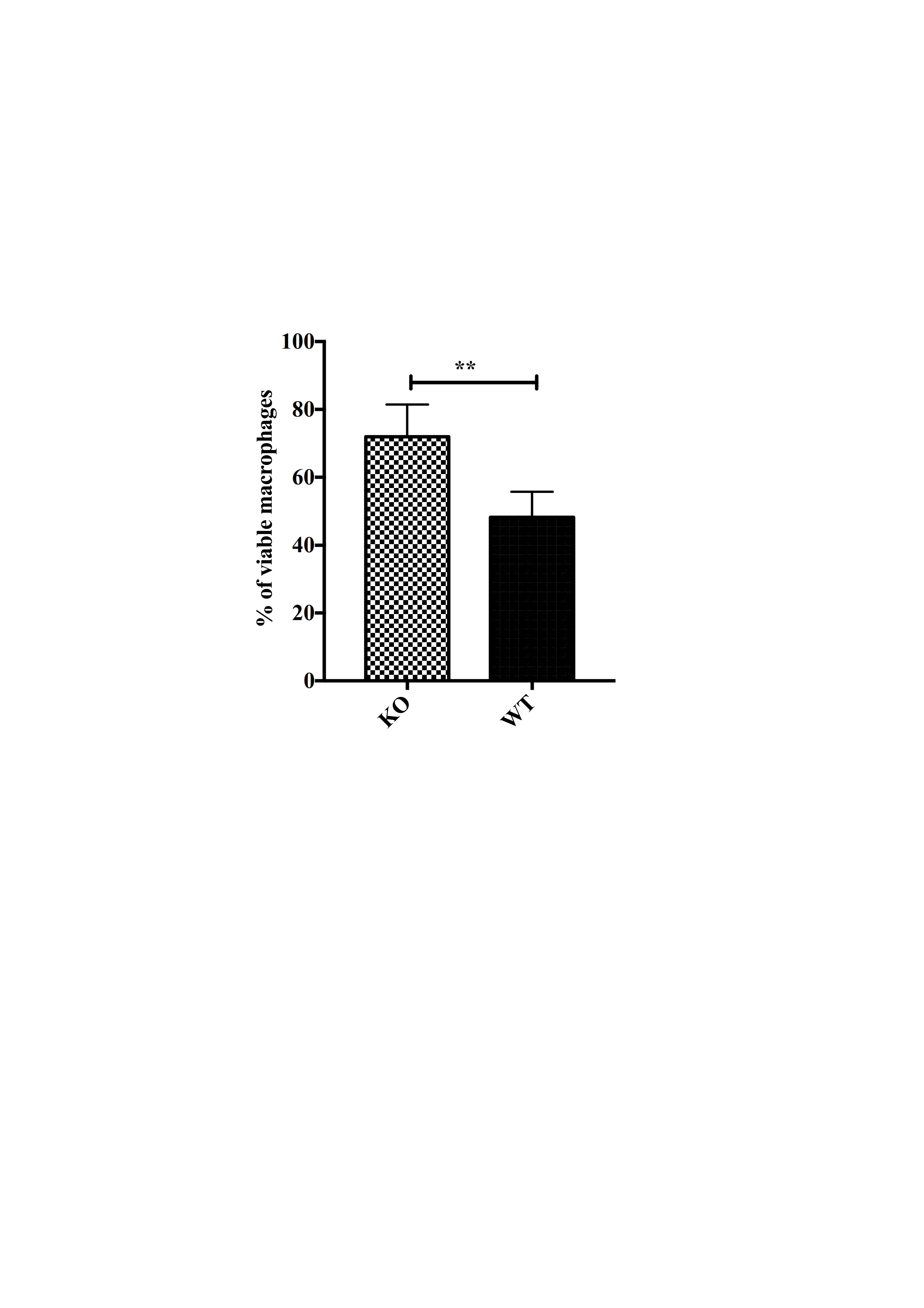

Supplement: Supplementary Figure 3 — Viability of KO macrophages after 7 days of M.tb infection. Lactate dehydrogenase (LDH) assay was performed and percentage of viable macrophages were calculated. Data are mean ± SEM of three independent experiments. **p < 0.005. [file Image_3.jpeg]

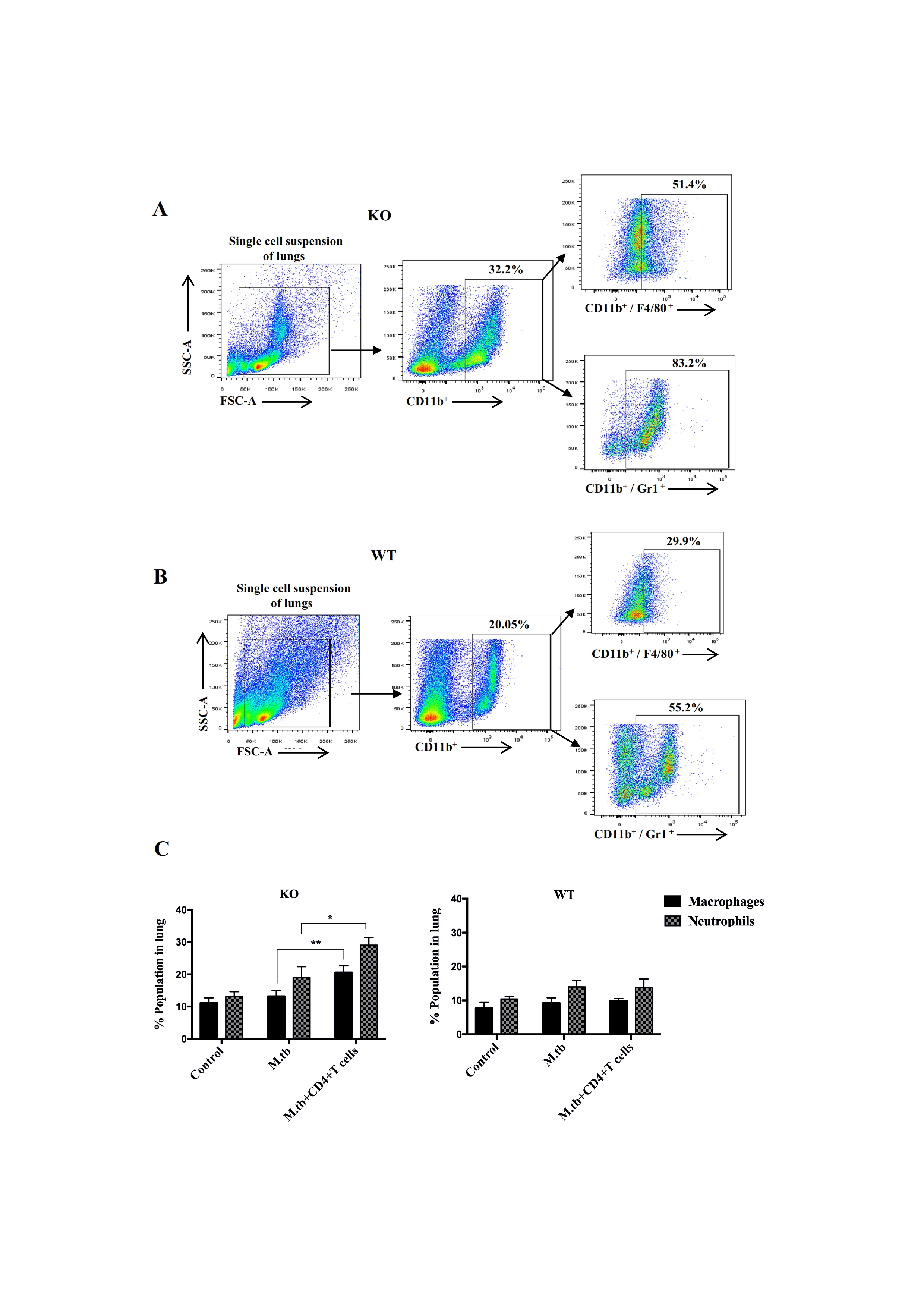

Supplement: Supplementary Figure 4 — Infiltration of macrophages and neutrophils in M.tb infected TCRβ−/− and WT mice lungs. Single cell suspension was prepared from lungs of different groups of KO and WT mice. Cells were stained with anti-CD11b, anti-F4/80 and anti-Gr1 fluorescent antibodies to analyze macrophages and neutrophils populations by flow cytometry. Representative flow cytometry plots of are presented, (A) M.tb infected KO mice lung single cell suspension after CD4+ T cells transfer, (B) M.tb infected WT mice lung single cell suspension after CD4+ T cells transfer are presented. Macrophages are F4/80+ and CD11b+ double positive. Neutrophils are Gr1+and CD11b+double positive. (C) Percentage population of macrophages and neutrophils in lungs of different groups of KO and WT mice are shown. [file Image_4.jpeg]

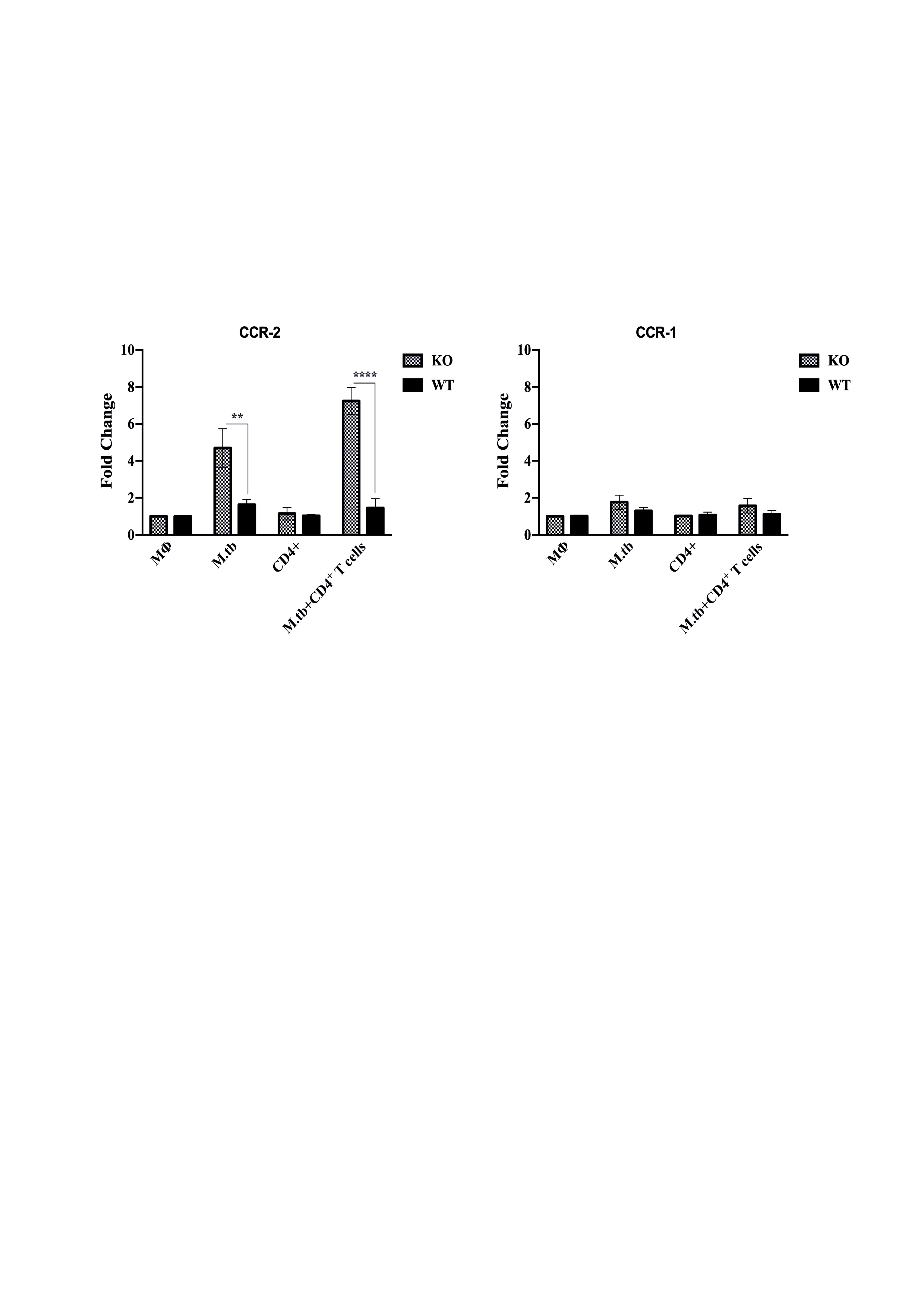

Supplement: Supplementary Figure 5 — mRNA expression of chemokine receptors in lung macrophages. mRNA expression of chemokine receptors was evaluated in macrophages from lungs of M.tb infected and uninfected TCRβ−/− and WT mice 15days post CD4+ T cells transfer. Data are mean ± SEM of three independent sets of experiments. Analysis was done using two-way ANOVA. **p < 0.005, ****p<0.00005. [file Image_5.jpeg]

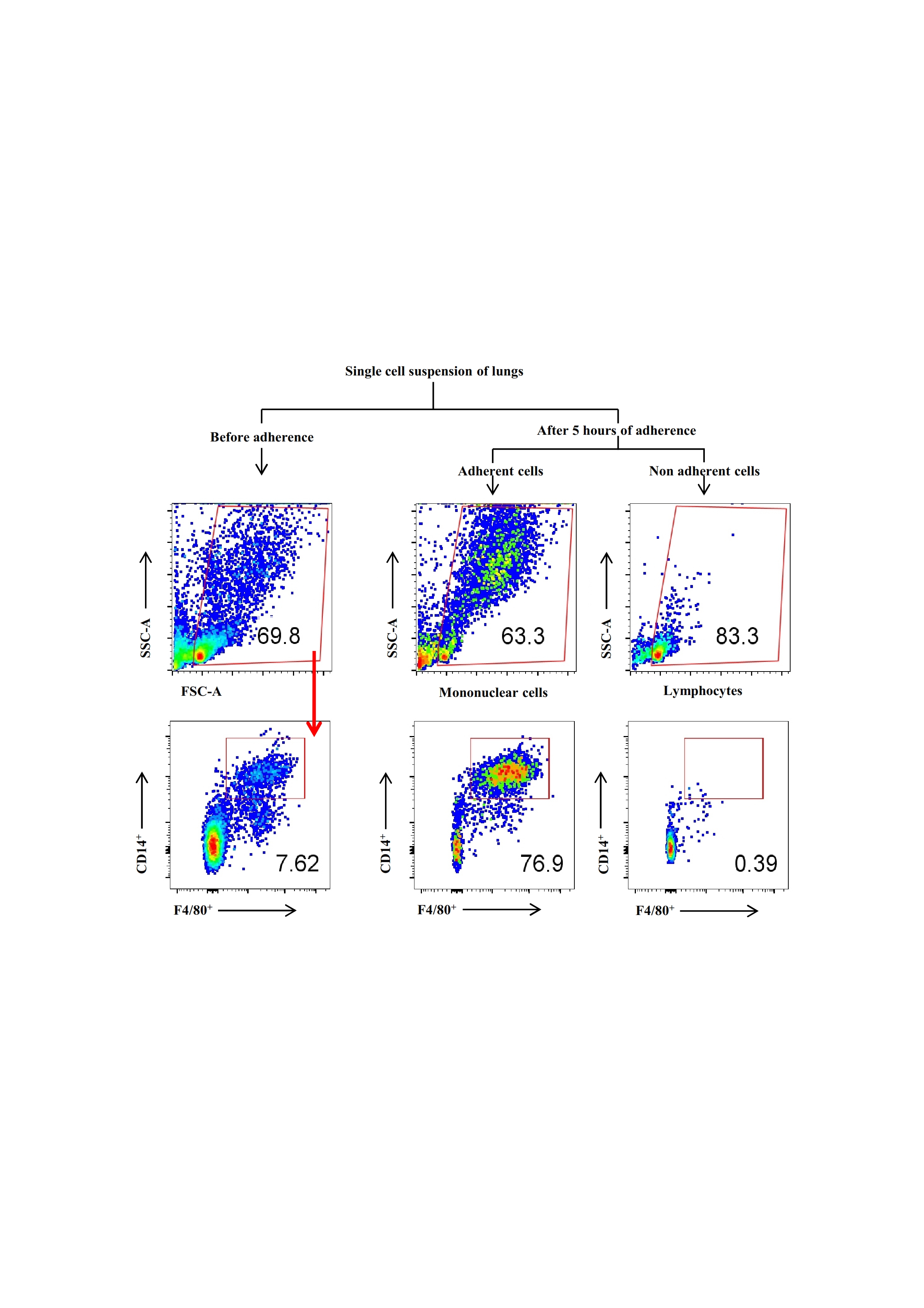

Supplement: Supplementary Figure 6 — Purity analysis of lung macrophages for mRNA isolation. Single cell suspension was prepared from lungs and allowed to adhere in culture flask for 5 hours; non-adherent cells were removed by washing twice with PBS. Purity of these cells were determined by flow cytometry. Cells were stained with anti-CD11b, anti-F4/80 and anti- CD14 fluorescent antibodies to analyze macrophages population. [file Image_6.jpeg]
